# How stable is lung function in patients with stable chronic obstructive pulmonary disease when monitored using a telehealth system? A longitudinal and home-based study

**DOI:** 10.1186/s12911-020-1103-6

**Published:** 2020-05-12

**Authors:** Åsa Holmner, Fredrik Öhberg, Urban Wiklund, Eva Bergmann, Anders Blomberg, Karin Wadell

**Affiliations:** 1 Department of Radiation Sciences, Biomedical Engineering, Umeå University, Umeå, Sweden; 2 Department of Epidemiology and Global Health, Umeå University, Umeå, Sweden; 3 Department of Public Health and Clinical Medicine, Section of Medicine, Umeå University, Umeå, Sweden; 4 Department of Community Medicine and Rehabilitation, Physiotherapy, Umeå University, Umeå, Sweden

**Keywords:** COPD, Spirometry, Home-monitoring, Long-term variations, Exacerbations, Telehealth

## Abstract

**Background:**

Many telehealth systems have been designed to identify signs of exacerbations in patients with chronic obstructive pulmonary disease (COPD), but few previous studies have reported the nature of recorded lung function data and what variations to expect in this group of individuals. The aim of the study was to evaluate the nature of individual diurnal, day-to-day and long-term variation in important prognostic markers of COPD exacerbations by employing a telehealth system developed in-house.

**Methods:**

Eight women and five men with COPD performed measurements (spirometry, pulse oximetry and the COPD assessment test (CAT)) three times per week for 4–6 months using the telehealth system. Short-term and long-term individual variations were assessed using the relative density and weekly means respectively. Quality of the spirometry measurements (forced expiratory volume in one second (FEV_1_) and inspiratory capacity (IC)) was assessed employing the criteria of American Thoracic Society (ATS)/European Respiratory Society (ERS) guidelines.

**Results:**

Close to 1100 measurements of both FEV_1_ and IC were performed during a total of 240 patient weeks. The two standard deviation ranges for intra-individual short-term variation were approximately ±210 mL and ± 350 mL for FEV_1_ and IC respectively. In long-term, spirometry values increased and decreased without notable changes in symptoms as reported by CAT, although it was unusual with a decrease of more than 50 mL per measurement of FEV_1_ between three consecutive measurement days. No exacerbation occurred. There was a moderate to strong positive correlation between FEV_1_ and IC, but weak or absent correlation with the other prognostic markers in the majority of the participants.

**Conclusions:**

Although FEV_1_ and IC varied within a noticeable range, no corresponding change in symptoms occurred. Therefore, this study reveals important and, to our knowledge, previously not reported information about short and long-term variability in prognostic markers in stable patients with COPD. The present data are of significance when defining criteria for detecting exacerbations using telehealth strategies.

## Background

Chronic obstructive pulmonary disease (COPD) is one of the most prevalent, costly and deadly non-communicable disease [1, 2] and the importance of effective COPD management has received a lot of attention. Spirometry (i.e. forced expiratory volume in one second (FEV_1_) and forced vital capacity (FVC)) is used for diagnosis and disease monitoring and these parameters are, by definition, seen as stable as long as the patients are in a stable state of the disease [3]. A major cause of hospitalization in COPD is acute exacerbations (AE), defined as a worsening of the patient’s respiratory symptoms that is beyond normal day-to-day variation [3]. AEs contribute significantly to healthcare consumption, hence the costs for COPD care, and patients with two or more exacerbations per year have a poor prognostic outcome [3, 4]. Regular monitoring of vital signs, physiological parameters or symptoms using telemonitoring or telehealth (TH) approaches has been proposed as a strategy for supporting early detection of exacerbations, allowing prompt initiation of treatment and faster recovery [5–7]. However, to be able to use these monitored physiological parameters as prognostic markers it is important to be aware of their inherent short and long-term variation, and their relation to clinically relevant symptoms, when recorded in a real-life situation.

Whilst the existing evidence is ambiguous, several TH studies have reported positive outcomes on hospital admissions and exacerbation frequency in COPD [8, 9]. Other studies report improved symptom control, functional status [10], self-management capacity [11] and health-related quality of life [12] compared to controls. There are also studies that have gone further and identified promising markers of COPD exacerbations [13–15], although some demonstrate poor predictive performance or even poor clinical outcomes [16–18]. Other studies have failed to draw any conclusions at all on the predictive performance of monitored parameters but, nonetheless, generated a positive outcome in terms of a reduction in exacerbation frequency [19]. A recent review including 29 telemonitoring studies on COPD, confirm these conflicting results with an almost equal distribution of improvements and no improvements in patient outcomes in the studies [20]. Common to most studies, however, is that they fail to report the nature of recorded data, e.g. with respect to differences within and between individuals, diurnal or day-to-day variations, frequency of outliers, long-term stability or variability and individual differences in adherence or learning effects. These data are needed to be able to judge the feasibility of a TH approach and to identify individuals suitable for enrolment in such a program. As it is well known that COPD is a heterogeneous disease, it is critical to take the individual at the starting point when designing TH programs and interventions. This knowledge-gap is the point of departure of the present study.

The aim of the study was to evaluate the nature of individual diurnal, day-to-day and long-term variation in spirometry measurements (FEV_1_ and inspiratory capacity (IC)), and their co-variation with other important prognostic markers of COPD exacerbations (oxygen saturation (SpO_2_) and symptoms measured with COPD assessment test (CAT)) by employing a telehealth system developed in-house.

## Methods

### Mobile home monitoring system

Data collection was enabled through a mobile health monitoring system developed in-house and described elsewhere [21]. In summary, the system consisted of a spirometer (SpiroTube Mobile Edition, Thor Laboratories, Hungary) connected via Bluetooth or cable to a tablet computer (Acer Iconia Tab W501P, 2011), providing instructions for spirometry manoeuvres and an electronic version of the CAT [22]. To permit recording saturation, another potential prognostic marker, the system was upgraded prior to the present study with an application for a Bluetooth-connected pulse oximeter (Nonin WristOx model 3250, Nonin Medical Inc., Plymouth, MN). The final version of the system thus consisted of a tablet computer with three applications, one for the CAT, one for pulse oximetry and one for a spirometry protocol. Unprocessed data were stored locally until a mobile broadband connection was established and thereafter automatically uploaded to a secure server for further processing.

### Participants and study design

During the years 2013–2015, consecutive participants with moderate to very severe COPD according to Global initiative for chronic Obstructive lung disease (GOLD) guidelines [3] were recruited from the Department of Medicine, Division of Respiratory Medicine and Allergy, University hospital of Umeå, Sweden. Care was taken to include patients with a history of exacerbations. After obtaining informed consent, participants completed an outpatient visit that included standard medical examination, chest x-ray, static and dynamic spirometry, venous blood tests and six-minute walk tests [23]. Baseline value of the participant’s perceived respiratory impairment was obtained using the modified Medical Research Council (mMRC) dyspnoea scale [24].

The study had a pragmatic approach and provided a minimum of individual education prior to enrolment as well as limited support during the trial. Before returning home with the equipment, participants received a short instruction (15–20 min) to the home monitoring system and were asked to execute the study protocol with guidance from a researcher involved in the study. The protocol implemented on the tablet computer was executed in the following order: First, the participant was asked to answer the eight questions of the CAT. This test was followed by pulse oximetry for 1 min. Finally, the participant executed a spirometry protocol consisting of a minimum of three IC manoeuvres, followed by a minimum of three FEV_6_ manoeuvres to enable calculation of FEV_1_. It was possible to repeat a manoeuvre if needed. During spirometry manoeuvres, participants were instructed to sit in an upright position with a 90-degree flexion in hips and knees and to use a nose clip.

The first week of the study, the participants performed the above-mentioned protocol twice a day. Thereafter, the protocol was repeated morning and evening 3 days a week for 4–6 months. The frequency of 3 days per week was chosen to closely monitor potential variation, yet achieve a relevant compliance to the procedure. Participants inhaled subscribed medications before the morning spirometry procedures. When prescribed inhalation therapy bi daily, the evening spirometry was carried out before drug administration. Technical support was provided during the study, but the participants did not receive any medical follow-up based on the recorded data. Participants were thus aware that they should contact their usual healthcare provider if they needed medical attention.

After completion of the study, the participants performed another outpatient visit, including one 6-min walk test, dynamic spirometry and assessment of perceived respiratory impairment (mMRC).

### Data management & statistical analysis

During ongoing measurements, oxygen saturation, heart rate and CAT scores were displayed to the participants. During the FEV_1_ and IC manoeuvres, visual cues indicating airflow direction were shown to the user, but no results from the spirometry were presented on the device. After the recording, data were uploaded to a server at the hospital for further analyses. More details regarding the recording and processing of the recorded lung function signals can be found in the Supplementary Materials. All data processing and statistics were performed using MATLAB (R2016b, MathWorks, Natick, Massachusetts, USA) and R software (version 3.3.3).

Feasibility of home spirometry was judged by estimating the overall adherence to the study protocol and based on the quality of the FEV_1_ and IC measurements, as defined by the American Thoracic Society (ATS)/European Respiratory Society (ERS) guidelines [25]. Adherence to the study protocol was defined as the ratio between the number of times the spirometry protocol was executed and the total number of scheduled measurements for the whole study period, i.e., six times a week. The first 2 weeks of the study were classified as a learning phase and thus excluded from the analysis, as were shorter interruptions owing to breaks that were agreed upon or unforeseen system maintenance.

The quality of each breathing manoeuvre was visually inspected by one investigator. Occasionally, patients had performed more than three efforts to obtain three tests that they considered acceptable, but the three best measurements were selected for calculation of FEV_1_ and IC, according to the ATS/ERS guidelines [25].

The adherence to the ATS/ERS guidelines was evaluated by calculating the difference between the largest and second largest FEV_1_. If the difference was < 0.150 L, the test was considered acceptable. The difference was also compared with the threshold 0.100 L, which is the corresponding threshold for participants with a FVC below 1.0 L. In this study, all participants presented with FVC > 1.0 L at the baseline post-bronchodilator examination. Pearson’s correlation coefficient was calculated to assess the agreement between the different measured recordings.

For IC, the coefficient of variation (CV) was determined between the three acceptable manoeuvres. Since the mean CV in chronic airflow obstruction has been found to be approximately 5% [26], the quality of the IC data was evaluated by comparing CV with the thresholds 5, 8 and 10%, respectively.

The largest FEV_1_ and the average IC from each test occasion were used in further analyses. All recordings (heart rate, SpO_2_, FEV_1_, IC and CAT) were thereafter compiled and analysed on group level as well as on individual level with respect to the individual diurnal, day-to-day and long-term variations.

An analysis of factors affecting the different outcome measures on a group level was performed using linear mixed models (R-package ‘lme4’ version 1.1.12), where the response measure was set to FEV_1_, IC, SpO_2_ and CAT. The model included the fixed effects of sex (male and female), measurement period (beginning, mid and late) and time of day: morning (AM) and afternoon/evening (PM). The included random effect was a random intercept on participant identity.

The long-term variation was assessed by calculating the mean of all measurements in the same week. The short-term variation in each participant, including the diurnal variation, was evaluated by estimating the relative density of changes between successive measurements and between measurement performed at the same time of the day (morning or evening). Additionally, the presence of periods with successive decreases between measurements was determined by defining a significant change as a decrease > 50 ml in FEV_1_ or IC between two consecutive measurements. Thus, the total decrease was > 100 ml between the first and third of the successive measurements. We only considered changes between measurements from the same part of the day, i.e., from morning-to-morning or evening-to-evening, respectively. In addition, if a period with successive decreases over 2 days or more was found, FEV_1_ or IC was considered as unchanged if the next value increased with less than 50 ml. As soon as the next value increased > 50 ml from the previous value, the period with successive decreases was considered to have ended.

## Results

### Study population

Twenty-five participants fulfilling the inclusion criteria were asked for participation during the two-year inclusion period. Thirteen accepted to take part, and were included in the study. The recruited study population consisted of a heterogeneous group of participants represented by eight women and five men aged 48 to 79 years. The participants had a BMI ranging from 15.7–33.7, a FEV_1_% predicted ranging from 21 to 68 and SpO_2_ at rest ranging from 90 to 100%. The thirteen participants were spirometrically graded as GOLD 2 *n* = 6; GOLD 3, *n* = 5; and GOLD 4, *n* = 2. Twelve participants were characterized as GOLD stage D and one participant as GOLD stage A. Eight of the thirteen participants had a history of exacerbations in the preceding 12 months and five of them had had more than one. All but one remained stable throughout the study and the only confirmed exacerbation happened during a period when the system was on maintenance; thus, it could not be accounted for in the analysis. Two participants (GOLD stage 3D) were excluded from all analyses because of technical failure of the device. This resulted in a loss of nearly all recorded data in one participant, and very few performed recordings in the other participant. Characteristics of the 11 included patients are presented in Table 1.

**Table 1:** Baseline characteristics of study subjects

Sex (f/m) 8/3	Baseline	
	Median	IQR
**Age**	67	59–72
**Height (m)**	1.66	1.59–1.76
**Weight (kg)**	65	59–87
**BMI**	26	21–30
**VC (L)**	2.70	2.6–3.9
**VC (% pred)**	112	89–119
**FVC (L)**	2.15	1.8–3.0
**FVC (% pred)**	80	75–82
**FEV**_**1**_**(L)**	1.01	0.9–1.7
**FEV**_**1**_**(% pred)**	53	36–63
**FEV%**	54	38–62
**IC (L)**	1.89	1.6–2.5
**IC (% pred)**	98	82–106
**6MWD (m)**^**a**^	387	335–479
**SpO**_2_**rest**	96	95–98
**mMRC**	2	1.5–4

*BMI* Body Mass Index, *VC* Vital Capacity, *FVC* Forced Vital Capacity, *FEV*_***1***_ Forced Expiratory Volume in one second, *IC* Inspiratory Capacity, *6MWT* 6 min walking test, *SpO*_*2*_ Oxygen saturation measured by pulse oximetry, *mMRC* modified Medical Research Council Dyspnea Scale, *IQR* Inter Quartile Range (25–75), *pred* predicted, ^*a*^ best of two tests

### Adherence to study protocol and quality of home spirometry

After the initial learning period of 14 days, the remaining 11 participants recorded a total of 1090 forced expiratory volume measurements and 1088 inspiratory capacity measurements that were used to calculate FEV_1_ and IC according to the ERS/ATS guidelines [25]. The average adherence to the study protocol was 90.6%, defined as the ratio between the total number of times the spirometry procedure was executed and the total number of scheduled procedures. That is, less than one in ten recordings was skipped or forgotten (reasons for not performing manoeuvres are unknown).

A complete set of three correct FEV_1_ manoeuvres was recorded on 1070 occasions (98.2%) when the spirometry protocol was executed. On 16 occasions (1.5%), two correct procedures were recorded and on four additional occasions only one correct manoeuvre was recorded (< 1%). The between-manoeuvre variability, based on the difference between the highest and second highest FEV_1_ value, was calculated for all occasions with at least two acceptable recordings and resulted in ≤150 ml on 97.7% occasions and ≤ 100 ml on 93.1% occasions.

Of the 1088 occasions when the IC manoeuvre was performed, three correct manoeuvres were performed on 1071 occasions (98.3%), enabling calculation of an IC value. On 16 occasions (1.5%), only two correct manoeuvres were recorded and on one additional occasion only one correct manoeuvre was recorded. The between manoeuvre variability, estimated by the coefficient of variation (CV) on all occasions with a minimum of two acceptable measurements, resulted in a CV ≤ 10% in 88.2% of the occasions, a CV ≤ 8% in 79.0% of the occasions and a CV ≤ 5% in 56.2%, i.e., slightly more than half of the occasions.

### Home vs lab measurements

After the initial learning period, spirometry, pulse oximetry and CAT scoring were executed twice daily, morning and evening, three times a week. Table 2 and Table 3 present spirometry data from the first and last months compared to the participants’ baseline values. FEV_1_ and IC from home monitoring are presented as the ratio between the value from home monitoring and the baseline value. The unsupervised measurements generally produced lower values of FEV_1_, although there were participants that managed to pace themselves well enough to match the lab results (e.g., six participants with FEV_1_(home)/FEV_1_(baseline) ≥0.9 in AM and/or PM measurements during the first month). Unsupervised IC values were generally lower than the lab values. A notable difference (> 100 ml) between morning and evening values were found in several participants in both FEV_1_ and IC during both the first and last month: e.g. in FEV_1_ from the first month in participants 5 and 11. The nature of this difference was, however, not consistent; some participants systematically recorded higher lung function values in the morning, whereas others reached higher values in the afternoon.

**Table 2:** Monthly averages of FEV_1_ compared to measurements at the baseline examination

**Subject**	**Predicted value (L)**	**Baseline (L)**	**Home**^**a**^**- First month**		**Home**^**a**^**- Last month**	
			**AM**	**PM**	**AM**	**PM**
1	1.70	0.85	0.82	0.79	0.92	0.85
2	3.48	1.01	0.54	0.77	0.58	0.77
3	2.43	0.85	0.97	0.99	0.99	1.04
4	1.79	0.95	0.84	0.91	0.75	0.84
5	1.89	0.7	1.03	0.83	1.10	0.84
6	1.95	1.21	0.81	0.79	0.83	0.78
7	2.89	1.76	0.86	0.90	0.87	0.90
8	2.57	0.54	0.89	0.99	0.84	0.97
9	3.00	2.04	0.97	0.89	0.81	0.79
10	2.55	1.68	0.66	0.63	0.67	0.65
11	3.25	2.05	0.88	0.77	0.76	0.77
Mean (SD)	2.50 (0.61)	1.24 (0.55)	0.84 (0.14)	0.84 (0.11)	0.83 (0.14)	0.84 (0.11)

Values of home measurements are expressed as the ratio Home/Baseline (^**a**^), where Home is the monthly average of AM and PM measurements, respectively

**Table 3:** Monthly averages of IC compared to measurements at the baseline examination

Subject	Predicted value (L)	Baseline (L)	Home^**a**^- First month		Home^**a**^ - Last month	
			AM	PM	AM	PM
1	1.67	1.64	0.87	0.88	0.84	0.83
2	2.58	1.65	0.70	0.90	0.74	0.96
3	2.03	1.58	0.83	0.82	0.87	0.91
4	1.77	1.89	0.81	0.83	0.75	0.83
5	1.52	1.47	0.84	0.76	0.94	0.81
6	1.78	1.37	0.79	0.80	0.87	0.87
7	2.58	2.22	0.58	0.62	0.79	0.81
8	2.35	2.33	0.74	0.77	0.78	0.83
9	3.05	3.29	0.65	0.63	0.58	0.57
10	2.50	2.62	0.72	0.68	0.65	0.60
11	3.34	3.57	0.94	0.90	0.98	0.95
Mean (SD)	2.29 (0.59)	2.15 (0.74)	0.77 (0.11)	0.78 (0.10)	0.80 (0.12)	0.82 (0.13)

Values of home measurements are expressed as the ratio Home/Baseline (^**a**^), where Home is the monthly average of AM and PM measurements, respectively

As shown in Table 4, there was a moderate to strong positive correlation between FEV_1_ and IC in 9 of 11 subjects. However, the correlation between spirometry measurements and the other two prognostic COPD markers were in general absent or weak, except that the last four included subjects presented with a moderate to strong negative correlation between IC and CAT.

**Table 4:** Correlation between prognostic markers of COPD exacerbations (spirometry, CAT and SpO_2_)

Subject	FEV_**1**_ vs IC	IC vs CAT	FEV_**1**_ vs CAT	IC vs SpO_**2**_	FEV_**1**_ vs SpO_**2**_
1	0.48	−0.04	−0.14	0.22	0.20
2	0.90	−0.15	−0.20	0.14	0.15
3	0.45	−0.06	−0.01	0.03	0.04
4	0.60	−0.19	−0.18	−0.01	0.07
5	0.66	0.03	0.08	− 0.16	0.07
6	0.44	−0.16	− 0.19	− 0.01	0.10
7	−0.03	− 0.13	0.13	− 0.19	0.09
8	0.42	−0.35	0.12	0.20	0.31
9	0.53	−0.47	− 0.41	−0.03	− 0.06
10	0.58	−0.42	−0.15	− 0.03	−0.28
11	0.29	−0.58	0.18	0.21	−0.08
Median (IQR)	0.48 (0.17)	−0.16 (0.32)	−0.14 (0.30)	− 0.01 (0.22)	0.07 (0.17)

Values are correlation coefficients. *FEV*_***1***_ Forced Expiratory Volume in one second, *IC* Inspiratory Capacity, *CAT* COPD Assessment Test, *SpO*_*2*_ Oxygen saturation measured by pulse oximetry, *IQR* Inter Quartile Range

### Diurnal, day-to-day and long-term variations in lung function

To evaluate the variability in the recordings on shorter timescales, which is of relevance for disease management and telehealth purposes, we calculated the intra-individual variation in FEV_1_ and IC between successive measurements and between weeks.

**Fig. 1:**
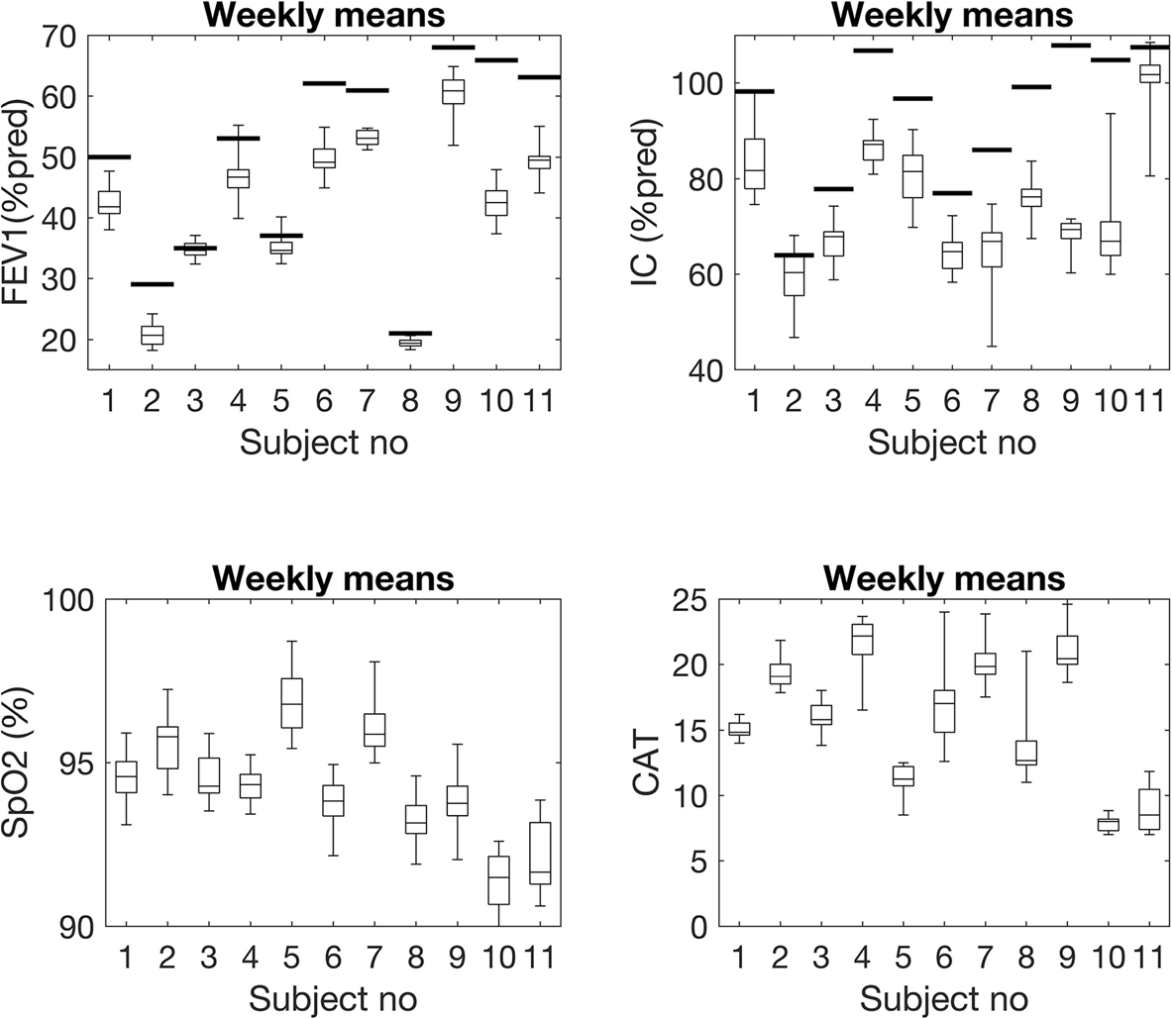
Weekly means of FEV_1_ (% of predicted), IC (% of predicted), SpO_2_ and CAT. Boxes show median and IQR, whiskers show range. Solid lines show the corresponding measurements from the baseline spirometry before the start of home monitoring. FEV_**1**_: Forced Expiratory Volume in one second, IC: Inspiratory Capacity, SpO_2_: Oxygen saturation measured by pulse oximetry, CAT: COPD Assessment Test

Figure 1 presents the distribution of weekly means in FEV_1_ (% predicted), IC (% predicted), SpO_2_, and CAT for all participants, overlaid on the corresponding lab values that were obtained at the time of enrolment in the study (see Table 1).

**Fig. 2:**
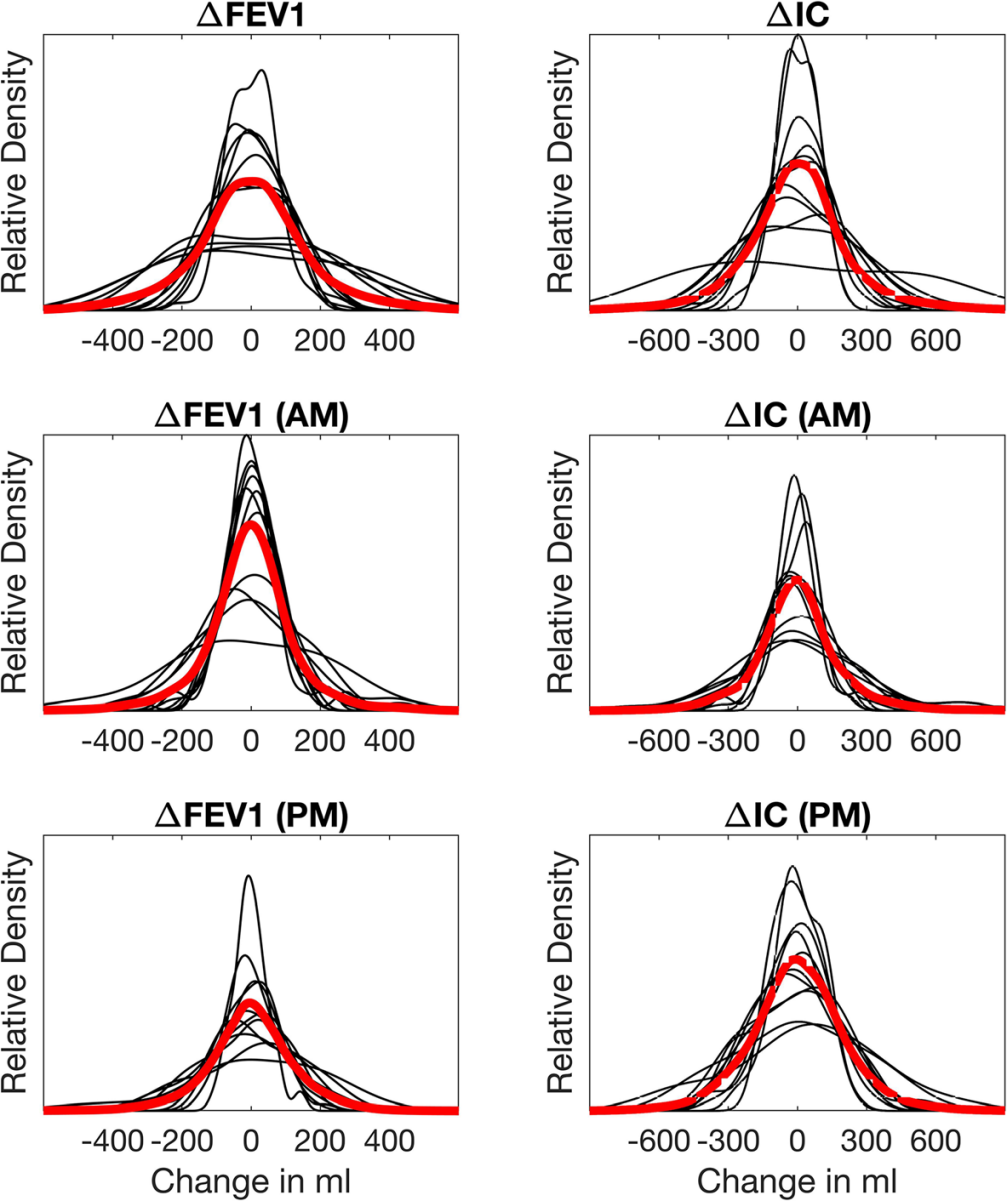
Relative density of the changes in of FEV_1_ (left) and IC (right) between measurements (top), between successive AM measurements (middle), and between successive PM measurements (bottom). Thin lines show individual curves. Red thick lines show the averaged density curve. FEV_**1**_: Forced Expiratory Volume in one second, IC: Inspiratory Capacity

Figure 2 shows the variation between consecutive measurements, which typically have a frequency of three weekly measurements (2–3 days between each measurement). The variability is presented using estimated density functions based on between-measurements changes in FEV_1_ and IC (percent of predicted values). There were marked differences in between-measurements variation between the participants, but also differences between individuals with respect to diurnal variation across several days. The variability between consecutive measurements (Fig. 2, top panels) showed two participants with a low and wide distribution due to a marked diurnal variation in IC, but distributions with two peaks also indicated a notable diurnal variation. Therefore, the distribution of consecutive AM-AM (middle panels) and PM-PM (bottom panels) are also presented in Fig. 2. The 2-SD range was − 211 to 210 ml for ∆FEV_1_ (AM) (− 8 to 8% for ∆FEV_1_ in %pred) and − 209 to 213 ml for ∆FEV_1_ (PM) (− 9 to 8% for ∆FEV_1_ in %pred), respectively. For IC, the 2-SD range was higher: − 286 to 351 ml for ∆IC (AM) (− 13 to 14% for ∆IC in %pred) and − 316 to 384 ml for ∆IC (PM) (− 15 to 15% for ∆IC in % pred), respectively.

When comparing measurements performed at the same time of the day, the analysis of successive decreases revealed a total of 34 periods in the 11 patients where FEV_1_ decreased with a total of > 50 ml over several consecutive measurements: 30 two-day periods and 4 three-day periods. After 29 of these 34 periods, FEV_1_ increased > 50 ml, whereas it remained unchanged 5 times. Moreover, in 30/34 of these periods, FEV_1_ was higher than the participant’s overall mean value before the onset of the decrease. There were only six two-day periods where FEV_1_ decreased with a total of > 100 ml over several consecutive measurements; in all these periods, FEV_1_ was higher than the overall mean value before the onset of the decrease. The same analysis for IC showed a total of 62 periods with successive decreases of > 50 ml from measurement-to-measurement: 51 two-day sequences and 11 three-day sequences. Of those, in 55/62 sequences (including all three-day periods), IC was higher than the mean before the onset of the decrease. There were 26 two-day periods and one three-day period where IC decreased with a total of > 100 ml over consecutive measurements; before the onset of the decrease IC was higher than the overall mean value in 23 of these periods.

On a group level, the linear mixed model analysis showed that FEV_1_ was significantly smaller during the middle period (− 0.02 L) compared to baseline. By the end of the measurement period, that difference has disappeared. In contrast, IC differed both in the middle and the end of the measurement period compared to baseline. In both cases, IC was 0.06 L higher compared to the baseline level. Also, there was a difference between AM and PM measures, where the measurements were 0.04 L higher in PM compared to AM. For SpO_2_, there was only a negligible difference (0.4% lower) in the final phase compared to baseline For CAT, the estimated values were lower; − 0.99 units and − 0.73 units below the middle and final stage respectively, compared with baseline.

## Discussion

As one of the first, this study reports important information about habitual variability in lung function, oxygen saturation and disease-related symptoms in individuals with COPD. The findings are based on more than one thousand unsupervised FEV_1_ and IC-recordings collected in a home environment. The study was originally designed to investigate whether a newly developed home monitoring system could detect early signs of exacerbation. Thus, care was taken to recruit patients with a recent history of exacerbations (10 out of 11 patients were GOLD D). Despite this focused selection, only one participant exacerbated during the whole study period. The reason for this is unclear, but might be a consequence of enhanced disease awareness, increased attention from health care, that the participants performed a set of breathing exercises regularly, or a combination of any of the above. This outcome per se is not unique, several telehealth studies have reported a reduction in exacerbation frequency and hospital admissions in telehealth groups compared to controls [9, 19, 27], which is one of the arguments for employing telehealth at all. For example, Segrelles-Calvo et al. demonstrated that monitoring of lung function, SpO_2_, heart rate and blood pressure with a TH system in patients on long-term home oxygen therapy significantly reduced the number of emergency visits, hospital admissions, length of hospital stay and need for non-invasive mechanical ventilation, compared to controls receiving conventional care [9]. These are all cost driving interventions suggesting a cost-benefit of the intervention. Tupper et al. also found improvements in health-related quality of life in patients equipped with a TH system recording symptoms, SpO_2_ and spirometry in addition to video consultations [12]. Unfortunately, there is very little information available on the nature of the telehealth data recorded in these studies and we have chosen to report data on short and long-term variations in measured indices; the inter and intra-measurement variability between measurements and adherence to the protocol. Moreover, we have chosen to report data also on an individual level, such as differences in diurnal and day to day variations in performance, and to highlight factors that are relevant to take into consideration when selecting suitable patients for employment of a TH system in ordinary care.

First, we estimated how well the 11 participants managed to perform the unsupervised spirometry by classifying all recorded spirometry manoeuvres either as correctly or incorrectly performed, as defined by the ATS/ERS guidelines [25]. Thereafter, the measurements were assessed with respect to repeatability, i.e., the within manoeuvre variability each time the spirometry protocol was executed, and with respect to adherence to the study protocol. Based on this assessment, we conclude that spirometry is indeed feasible to employ in a home environment. More than 98% of the occasions produced three acceptable manoeuvres with acceptable within manoeuvre variability, and adherence to the study protocol was judged to be very high, as more than 98% of scheduled routines were performed as planned. Compared to the pre-post FEV_1_ measurements, the self-paced measurements were typically lower although some managed to perform nearly as well at home as in the lab. Nonetheless, although slightly lower, the measurements at home remained stable and varied very little, particularly when comparing measurements performed the same time of the day over time. The same pattern was found in a study by Rodriguez-Roisin et al., who investigated change over time as a part of the WISDOM (Withdrawal of Inhaled Steroids During Optimized Bronchodilator Management) trial by having patients with severe to very severe COPD performing spirometry at home. Whilst they found a small difference with lower FEV_1_ values recorded at home compared with in the clinic, the change remained consistent and the data confirmed good agreement between measurements at home and at the clinic [28].

We did find cases in which FEV_1_ decreased during several consecutive measurements but without any signs of exacerbations or change in symptoms. A total of 34 periods were found where FEV_1_ decreased more than 100 ml between 3 and 4 consecutive measurements in total. A majority of those returned towards the “pre-decrease” values at the next measurement. Janssens et al. suggest that a change greater than 0.280–0.320 L is required to exceed the normal between-test variation [29]. However, the ATS/ERS guidelines report a minimal clinical important difference for FEV_1_ of 100 ml [30]. In addition, Redelmeier et al. state that changes within the normal variation may still represent a clinically meaningful result when accompanied by symptom changes [31].

Despite the lack of long-term trends in lung function, as indicated by the variation in weekly means of FEV_1_% predicted for each participant (Fig. 1), there is an evident difference in variability in spirometry both with respect to the variability between successive measurement and how the weekly means vary over time. The latter implies that a lung function value deviating from that of the previous week +/− the SD can still be within the normal range and does not necessarily imply a relevant change in lung function or an upcoming exacerbation, especially without corresponding change in symptoms, which was the case in this study.

Despite this variability in lung function measurements, we have drawn the conclusion that home spirometry is indeed feasible, though it should not be compared with measurements performed under supervision. The lung function values in the present study were consistently lower when performed at home compared to in clinic except for a few participants. Noted variations are thus not necessarily owing to poor spirometry performance. Compared to the pre-post values, the participants stayed consistent in their measurement results over time, though at a lower value (Fig. 1). None of the differences we found in the linear mixed model (on group level) were of clinical relevance. Another argument supporting this feasibility statement is that three complete and acceptable manoeuvres were performed in about 98% of the cases. In the UPLIFT study where the patients were followed with spirometry performed at clinic every sixth month for 4 years [29], they found that 86–92% of the manoeuvres were correctly performed and that a certain percentage of failed manoeuvres will occur, despite training and experience. The higher number of acceptable manoeuvres in the present study might be a result of more frequent measurements (i.e. three times per week).

The same consistent result was not found for inspiratory capacity (IC), a lung function measure that may indirectly reflect the severity of lung hyperinflation, which represents the fundamental origin of dyspnoea development in COPD. IC has thus been suggested to better reflect dyspnoea than FEV_1_ [32] and to be a stronger predictor of exacerbations when divided by the total lung capacity of the patient [33]. Therefore, IC was chosen as a routine measurement in the present study. However, this manoeuvre was found to be harder to perform correctly unsupervised compared to FEV_1_. Consequently, it was more difficult to interpret data from the IC-manoeuvres, making this parameter less suitable to measure in an unsupervised procedure. This might reflect that COPD patients in general are much more used to perform the FEV_1_ manoeuvres, even though they are more effort dependent. We still believe that IC might be a promising candidate for telehealth monitoring purposes as a few of the participants in the study showed a correlation between IC and symptoms, a correlation less prominent for FEV_1_ (Table 4).

### Clinical implications

Telehealth is considered to enable close monitoring of the patient in a real life situation as well as transferring data on a regular basis to the clinic. Home monitoring of patients with COPD has received increased attention the last years. Both to help patients with self-management strategies but also to be able to detect exacerbations and by preset alarms help decision making for healthcare professionals. Many telehealth systems have been designed to identify signs of COPD exacerbations, but few previous studies have reported the nature of recorded lung function data and what variations to expect in stable versus unstable patients. This is of relevance when interpreting results of home monitored lung function parameters. From a clinical perspective, it is also important to relate the variation in lung function to the spirometric COPD grade. However, this could not be done in this study due to the low number of participants.

### Study limitations

In this study, we used a pragmatic approach. The patients got limited support which may explain especially the results of the IC-measurements. Using some kind of guidance or feedback, the IC-manoeuvres might have been performed more like in clinic. It could easily be performed by including an instruction video as well as visual or audio feedback on the spirometry manoeuvre. This is in line with the recent review by Baroi et al. who concluded that remote respiratory assessments are feasible when combined with sufficient organizational back up [27]. However, considering the lack of feedback, we are surprised that the patients performed acceptable manoeuvres to such a large extent.

In the design of the study we aimed to recruit patients with frequent exacerbations. The majority of patients had a history of two or more exacerbations the previous year before inclusion. Though, the lack of exacerbation episodes during the study period makes it impossible to identify characteristics of individuals who might benefit the most of such a telehealth system at home and further studies are warranted.

## Conclusions

The study presents a large, intra-individual variation in lung function during the total of 240 patient weeks of monitoring. The variation was present even though neither exacerbations nor corresponding changes in symptoms occurred. This is important to take into consideration when monitoring patients at home in order to detect exacerbations. The multi-item telehealth system for home monitoring was feasible to employ in a home environment for patients with COPD, since more than 98% of the planned manoeuvres were conducted with acceptable within-manoeuvre variability. We also conclude that the IC-manoeuvres seemed to be more difficult than the forced expiratory manoeuvres to perform unsupervised.

## Supplementary information

**Additional file 1: Supplementary Materials.** Lung function measurements.

## Data Availability

The datasets used and/or analysed during the current study are available from the corresponding author on reasonable request.

## Abbreviations

AE: Acute exacerbations
AM: Morning
ATS: American Thoracic Society
CAT: COPD assessment test
COPD: Chronic obstructive pulmonary disease
CV: Coefficient of variation
ERS: European Respiratory Society
FEV_1_: Forced expiratory volume in one second
FEV_6_: Forced expiratory volume in six seconds
FVC: Forced vital capacity
GOLD: Global initiative for chronic Obstructive lung disease
IC: Inspiratory capacity
IQR: Inter Quartile Range
mMRC: Modified Medical Research Council Dyspnea scale
PM: Afternoon/evening
SpO_2_: Oxygen saturation
TH: Telehealth
