# Supplementary material for: How stable is lung function in patients with stable chronic obstructive pulmonary disease when monitored using a telehealth system? A longitudinal and home-based study
Source: BMC Med Inform Decis Mak. 2020 May 12;20:87. doi: 10.1186/s12911-020-1103-6 (PMC7218552; doi:10.1186/s12911-020-1103-6)
Supplement: Supplementary file 1 — Additional file 1: Supplementary Materials. Lung function measurements. [file 12911_2020_1103_MOESM1_ESM.docx]

**Supplementary Materials - Lung function measurements**

Figure S.1 shows the system used for telehealth monitoring. The airflow signal was sampled at 100 Hz and stored in the tablet computer, for later upload to a database at the hospital where all data analyses were performed.

The corresponding lung volume signal was determined by numerical integration of the flow signal. The Inspiratory capacity maneuver was analysed using a peak-valley detector, where the first three normal breaths were identified. Then, the maximum inspired volume was determined as shown in Figure S.2, from which inspired capacity (IC) was determined. Note that the subject either hold the breath after the last large inspiration or removed the mouth piece, which explain why the inhaled volume was nearly constant at the end of the recording.

Forced expiratory volume during one second (FEV1) was determined by first detecting the onset of maximum expiration using a threshold detector, and then by calculating the difference in expired lung volume one second later, as shown in Figure S.3.

­­


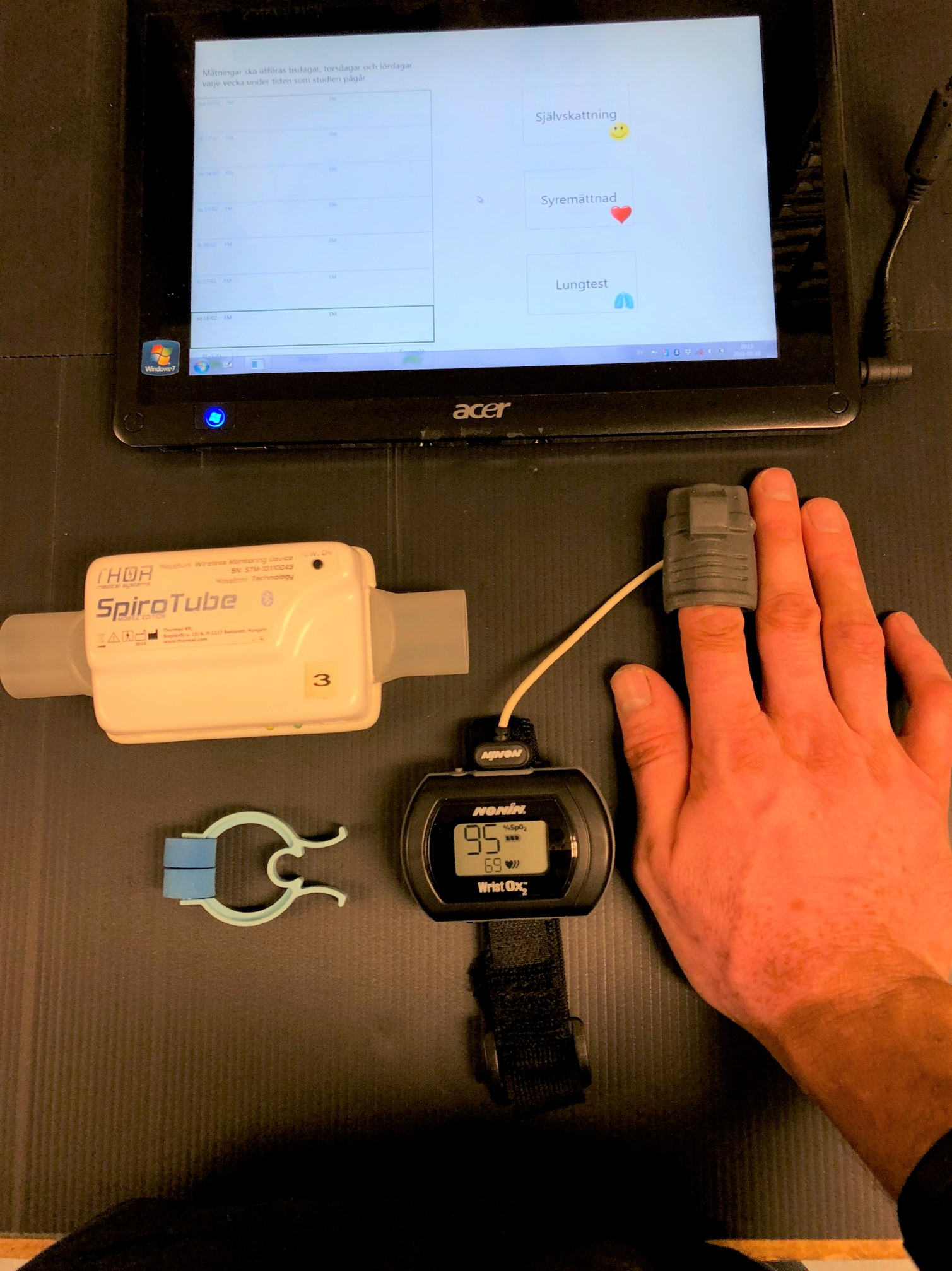


Figure S.1. The telehealth system, including a spirometer and a pulse oximeter both connected via Bluetooth to a tablet computer, providing instructions for spirometry maneuvers.

Figure S.2. Determination of inspiratory capacity in one of the subjects. Black circles correspond to end of expiration, whereas red circles correspond to end of inspiration.

Figure S.3. Determination of FEV1 in one of the subjects. Circles correspond to the onset of maximal expiration and the volume after one second, respectively.
